# Supplementary material for: Fine-mapping of prostate cancer susceptibility loci in a large meta-analysis identifies candidate causal variants
Source: Nat Commun. 2018 Jun 11;9:2256. doi: 10.1038/s41467-018-04109-8 (PMC5995836; doi:10.1038/s41467-018-04109-8)
Supplement: Supplementary file 1 — Supplementary Information [file 41467_2018_4109_MOESM1_ESM.pdf]

Supplementary Figure 1

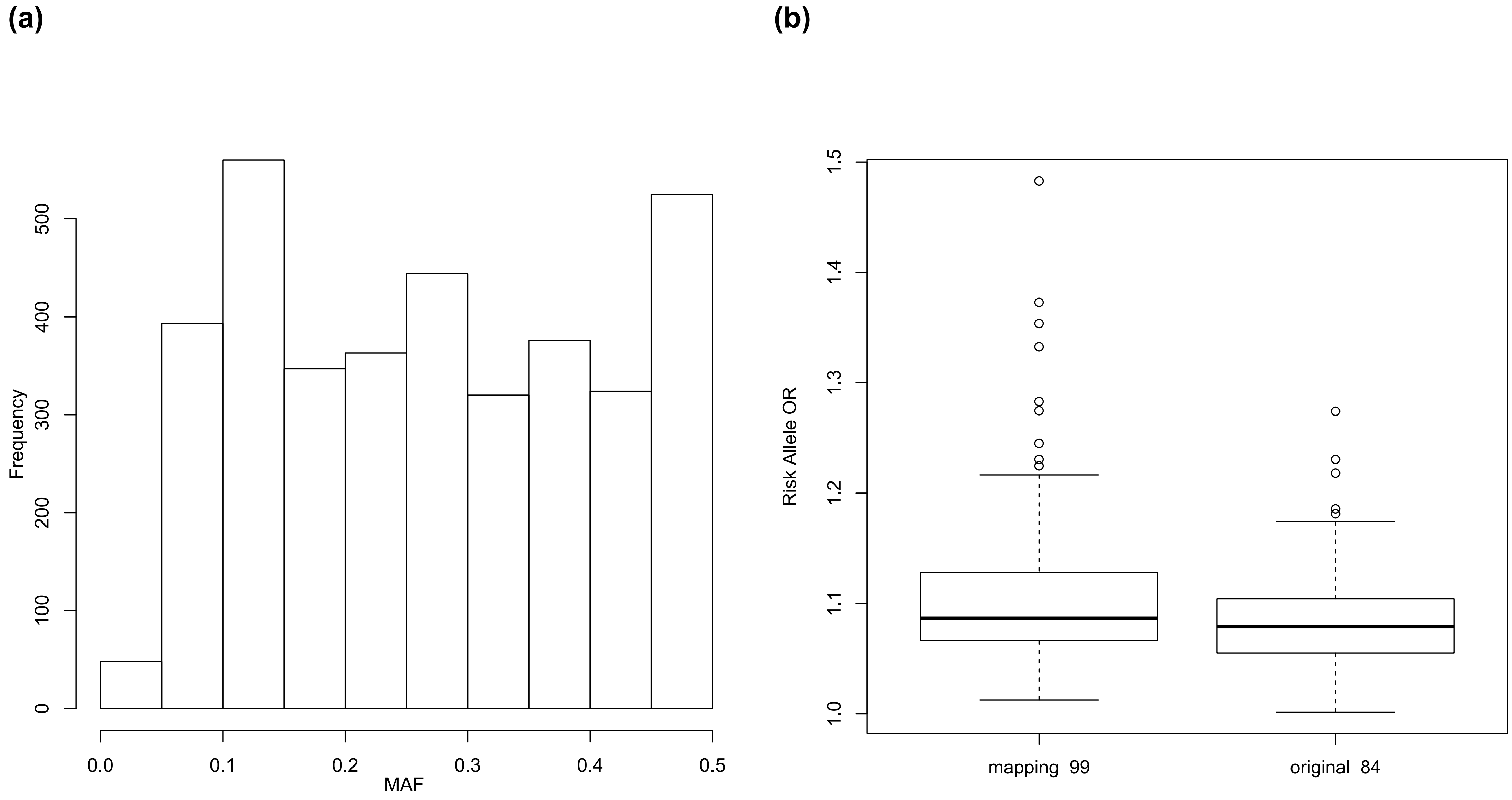

Supplementary Figure 1 – (a) Minor allele frequency distribution of variants within the 95% credible set selected by JAM. **(b)** Odds ratios of the 84 original GWAS tag SNPs replicated in Europeans relative to the 99 novel representative lead SNPs identified through fine-mapping. Single variants representing the 99 independent signals were established using the procedure described for familial relative risk calculation. The high odds ratio variant rs138213197 at *HOXB13* is omitted from this plot for greater clarity at the remaining regions with lower effect sizes. Box plot centre lines represent the median odds ratios for each variant set. Lower and upper hinges represent the first and third quartiles respectively, with whiskers denoting the largest and smallest values within 1.5 IQR (interquartile range) of the quartiles and outlying values plotted as individual points.

Supplementary Figure 2

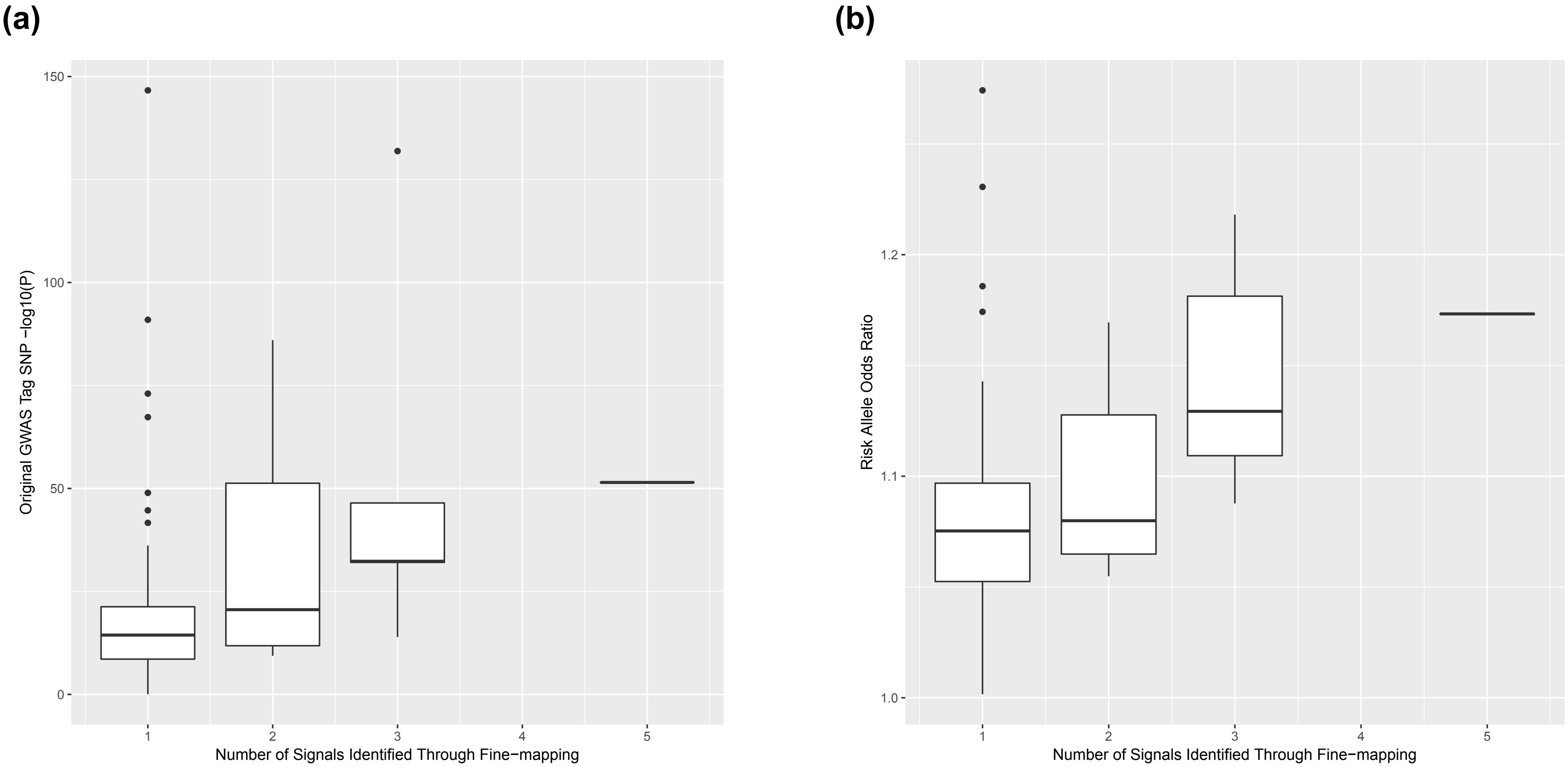

Supplementary Figure 2 – Comparison of the number of signals identified through subsequent Bayesian fine-mapping with **(a)** *P*-values and **(b)** Odds Ratios in the original meta-analysis for the 84 European replicated original GWAS tag SNPs.
The high odds ratio variant rs138213197 at *HOXB13* is omitted from these plots for greater clarity at the remaining regions with lower effect sizes. Box plot centre lines represent the median *P*-value or odds ratios for regions containing different numbers of independent signals. Lower and upper hinges represent the first and third quartiles respectively, with whiskers denoting the largest and smallest values within 1.5 IQR (interquartile range) of the quartiles and outlying values plotted as individual points.

Supplementary Figure 3

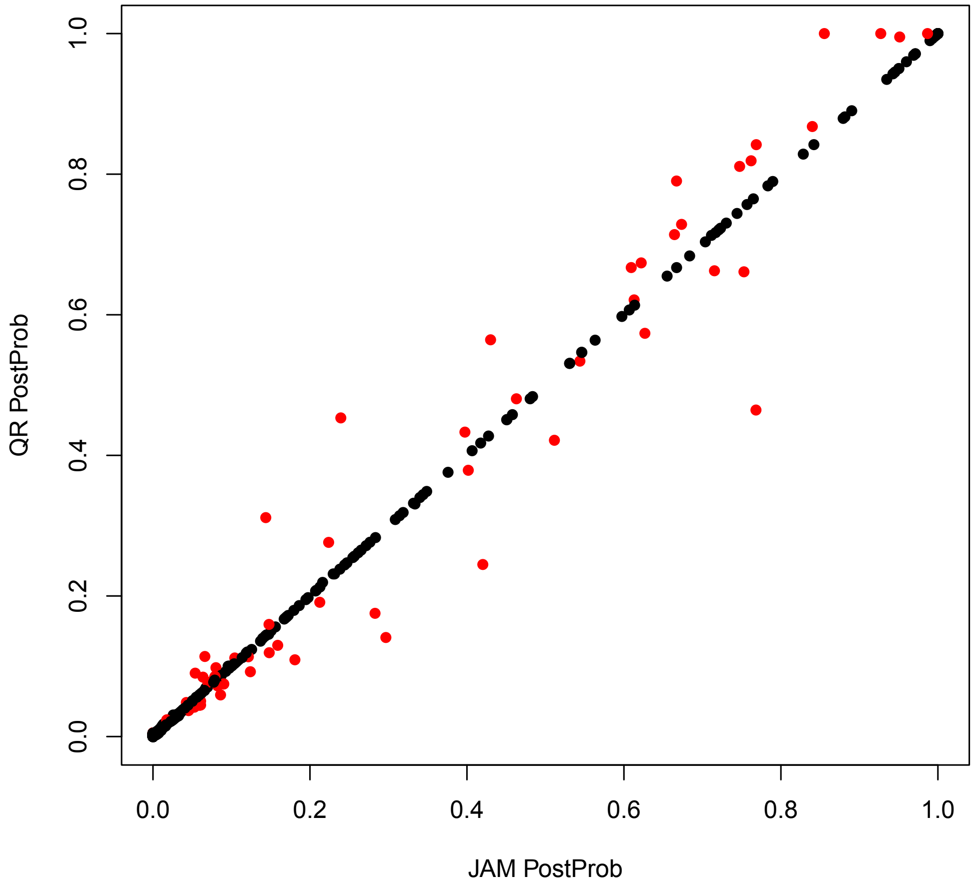

Supplementary Figure 3 – Overview of tag variant posterior probabilities recalculated during Quantile Regression across all 75 regions included in this analysis. Tags marked in red were adjusted by ΔPosterior probability$_{QR}$ ≥ ±0.005 based on annotations associated across the upper quantiles of the posterior probability distribution.

Supplementary Figure 4

(a)

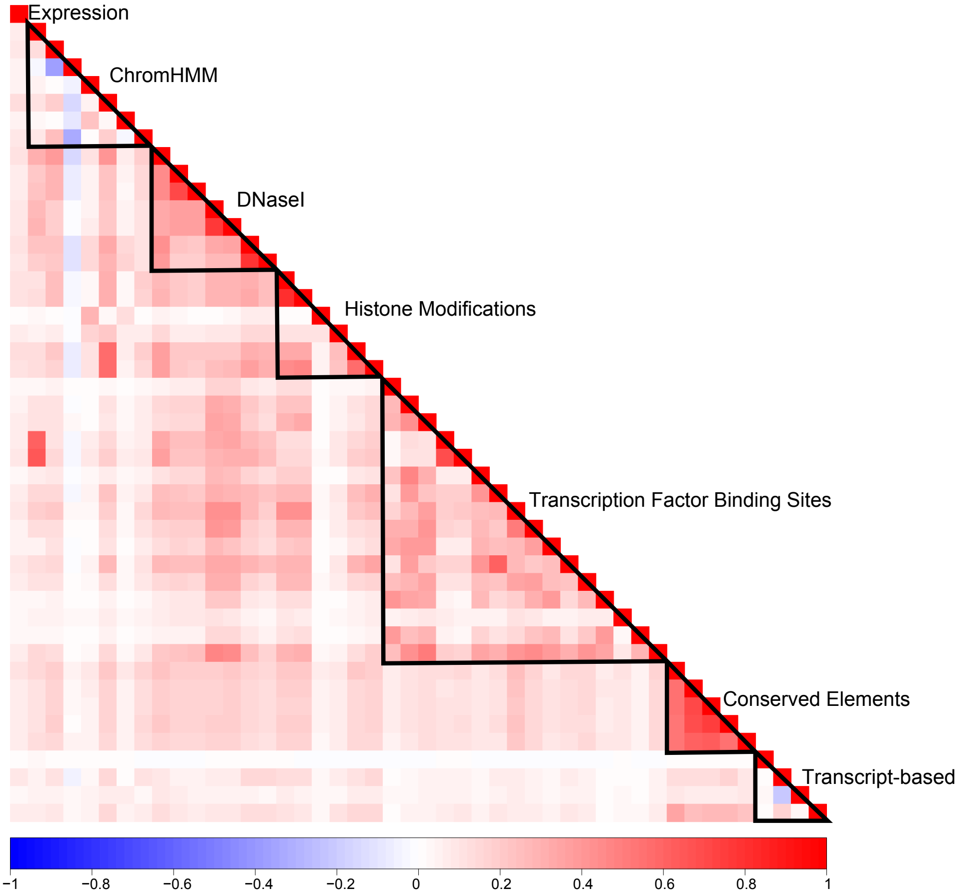

(b)

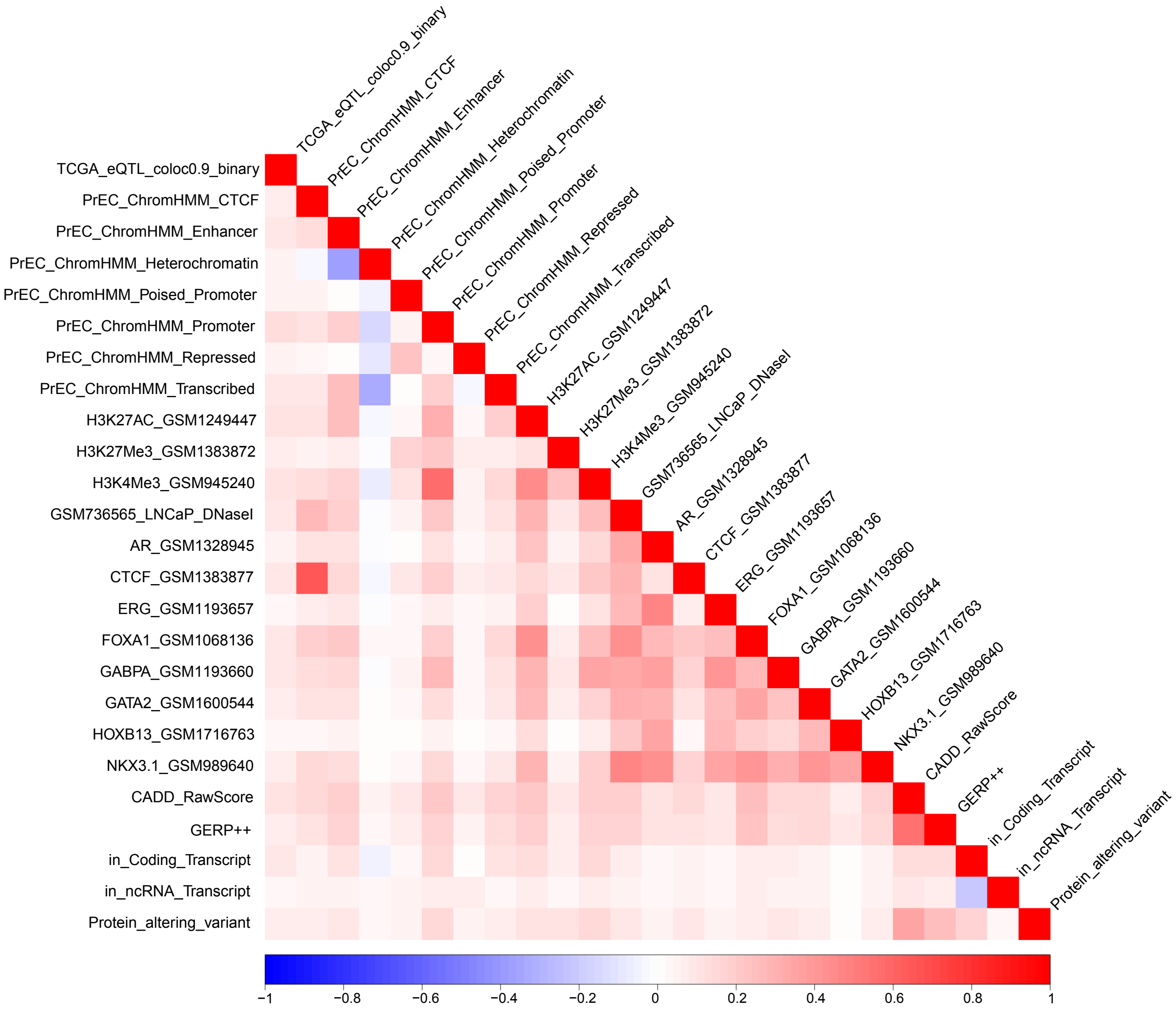

Supplementary Figure 4 – (a) Correlation between annotation categories is shown for priority pruner tags, which inherit annotations for all of their respective proxy variants. **(b)** Correlation between the individual annotations used in the Quantile Regression analysis, for which a single informative dataset was selected for each annotation group to avoid inclusion of datasets representing the same information class.

Supplementary Figure 5

(a)

	Estimate	Std. Error	z value	Pr(>|z|)	
(Intercept)	-5.23448	0.16680	-31.383	< 2e-16	***
PrEC_ChromHMM_CTCF	-0.83398	0.34150	-2.442	0.014603	*
PrEC_ChromHMM_Enhancer	-0.13724	0.14780	-0.929	0.353110	
PrEC_ChromHMM_Heterochromatin	-0.24523	0.16075	-1.526	0.127118	
PrEC_ChromHMM_Poised_Promoter	-0.20273	0.54338	-0.373	0.709086	
PrEC_ChromHMM_Promoter	-0.16644	0.22030	-0.756	0.449920	
PrEC_ChromHMM_Repressed	-0.03525	0.28774	-0.122	0.902507	
PrEC_ChromHMM_Transcribed	0.10251	0.15266	0.672	0.501890	
GSM736565_LNCaP_DNaseI	0.32281	0.20392	1.583	0.113414	
AR_GSM1328945	0.75119	0.27154	2.766	0.005667	**
CTCF_GSM1383877	0.05604	0.27992	0.200	0.841318	
ERG_GSM1193657	1.00400	0.27891	3.600	0.000319	***
FOXA1_GSM1068136	0.28245	0.17760	1.590	0.111752	
GABPA_GSM1193660	-0.35188	0.26055	-1.351	0.176844	
GATA2_GSM1600544	0.18173	0.23585	0.771	0.440981	
HOXB13_GSM1716763	0.22586	0.35916	0.629	0.529438	
NKX3.1_GSM989640	-0.06221	0.25740	-0.242	0.809021	
TCGA_eQTL_coloc0.9_binary	0.99863	0.12126	8.235	< 2e-16	***
CADD_RawScore	0.05195	0.07398	0.702	0.482577	
GERP++	0.25948	0.15431	1.682	0.092662	.
H3K27AC_GSM1249447	0.44695	0.18880	2.367	0.017921	*
H3K27Me3_GSM1383872	-0.51028	0.53817	-0.948	0.343042	
H3K4Me3_GSM945240	0.38463	0.23950	1.606	0.108285	
in_Coding_Transcript	0.43440	0.12286	3.536	0.000407	***
in_ncRNA_Transcript	-0.01336	0.16155	-0.083	0.934115	
Protein_altering_variant	0.10779	0.25930	0.416	0.677645	

(b)

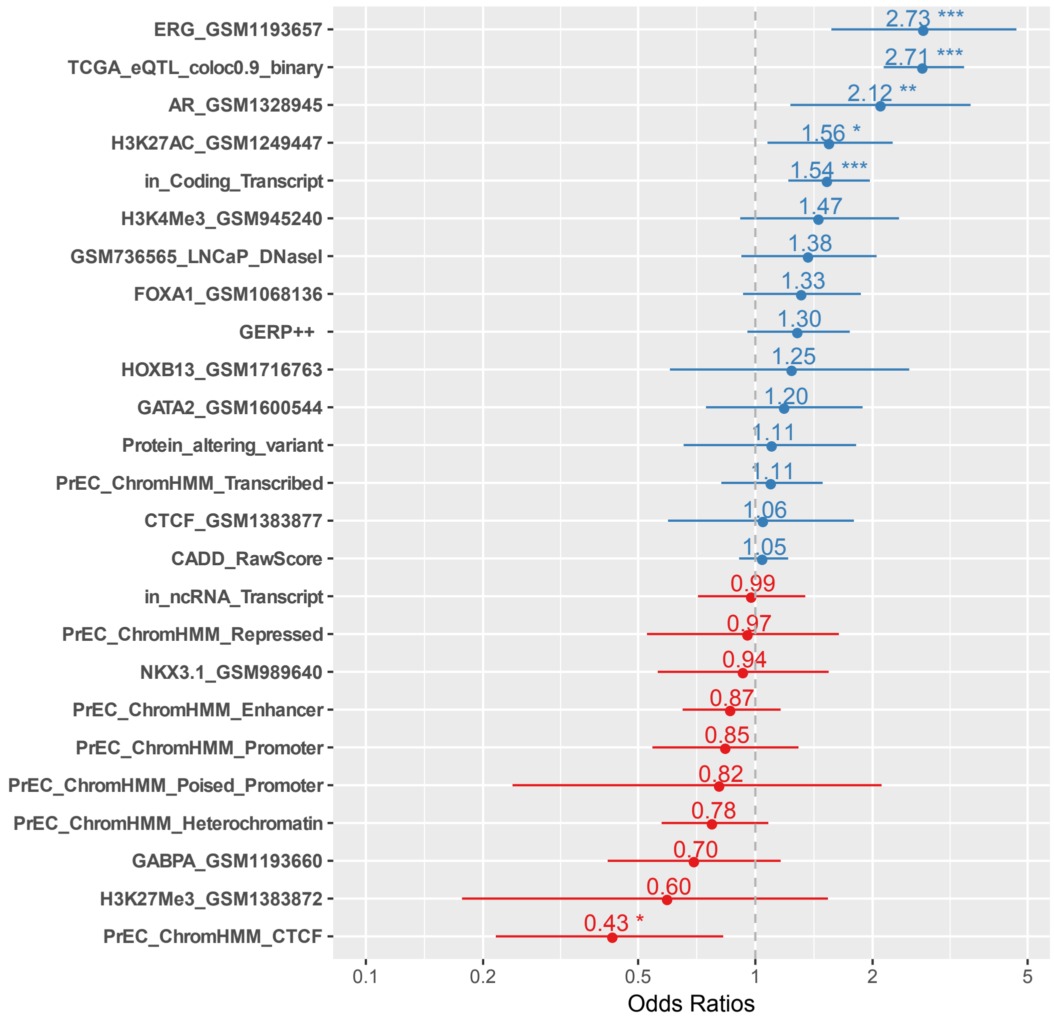

Supplementary Figure 5 – Results of logistic regression across the individual annotations used in the Quantile Regression analysis, for which a single informative dataset was selected for each annotation category to avoid inclusion of datasets representing the same information class. **(a)** Logistic regression model coefficient output. For each annotation feature covariate, variable coefficients ("Estimate") and standard errors ("Std. Error") are shown, alongside their corresponding z-statistic ("z value") and two-tailed *P*-values ("Pr(>|z|)"). **(b)** Dot plot of odds ratio estimates and 95% confidence intervals for each annotation feature in the logistic regression model. For both panels, single, double or triple star symbols denote annotation features nominally significant at $P < 0.05$, 0.01 and 0.001 thresholds respectively.

Supplementary Figure 6

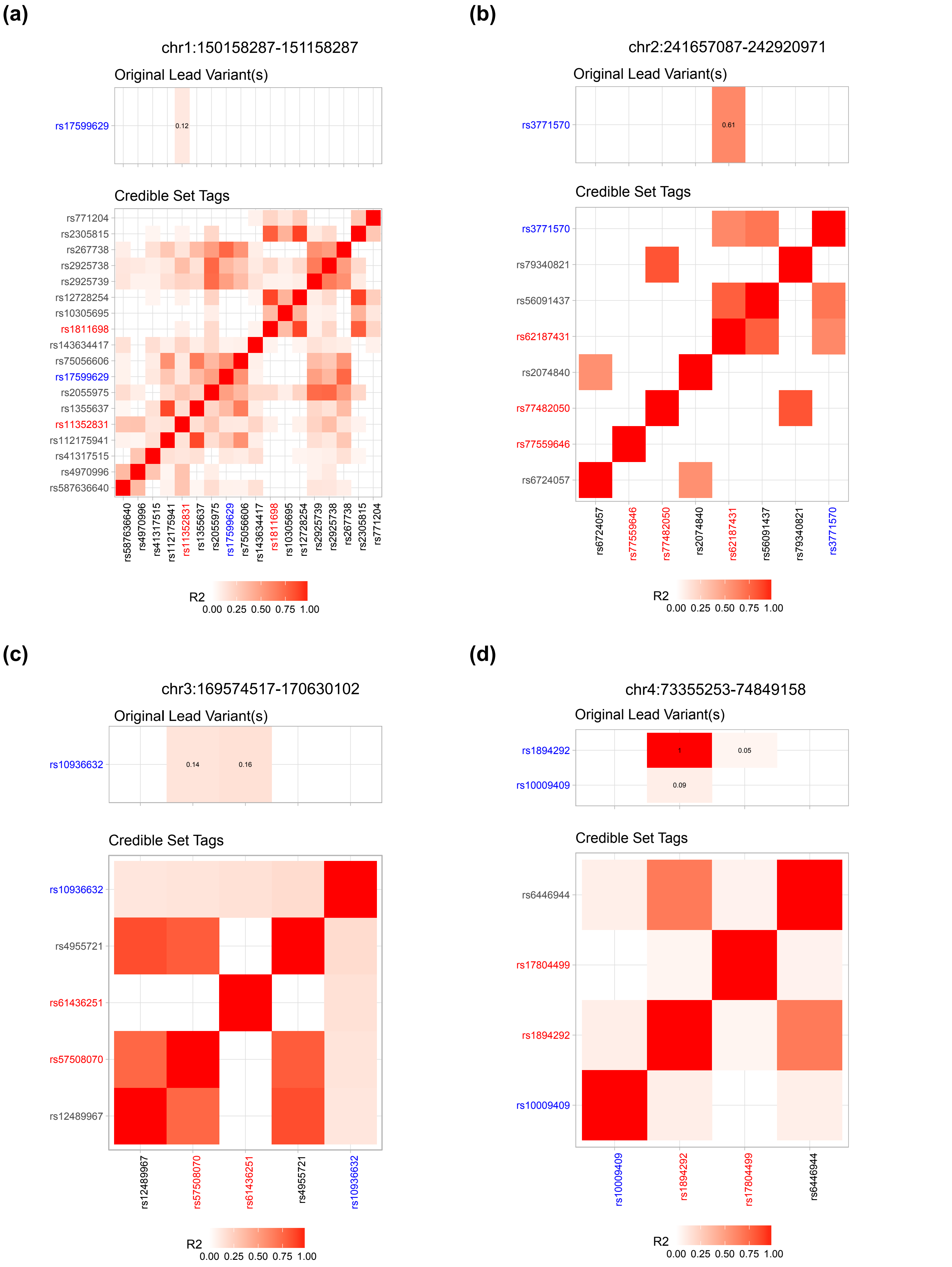

Supplementary Figure 6 (continued)

(e)

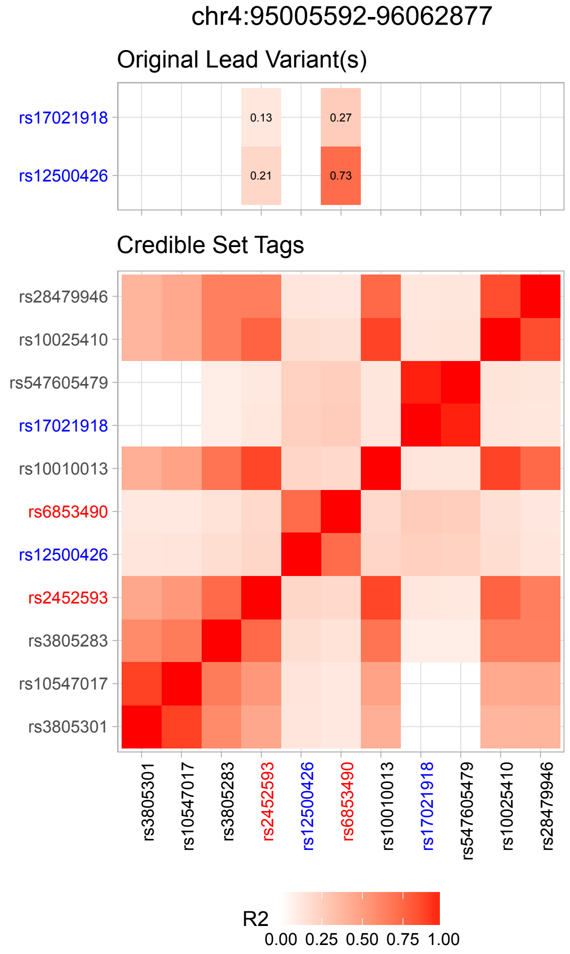

(f)

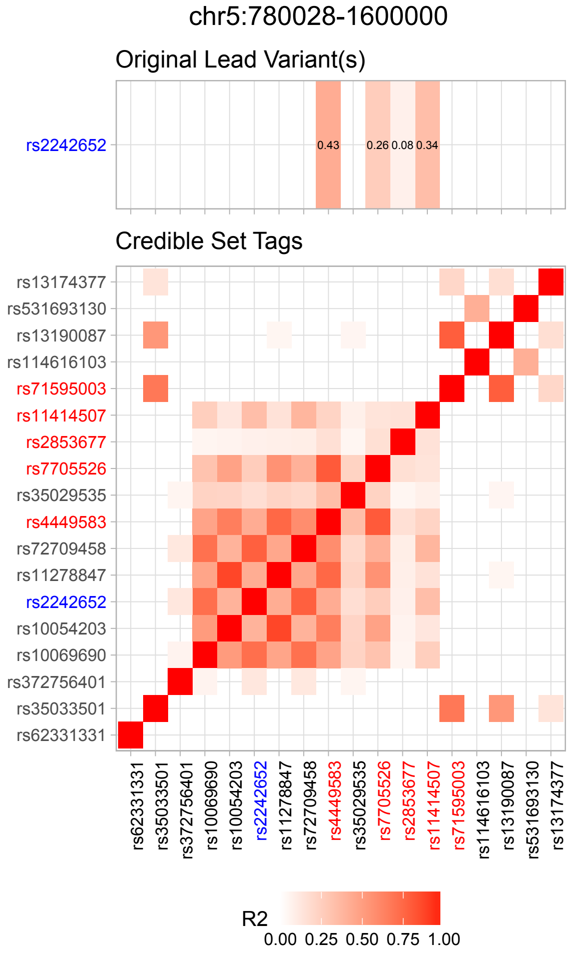

(g)

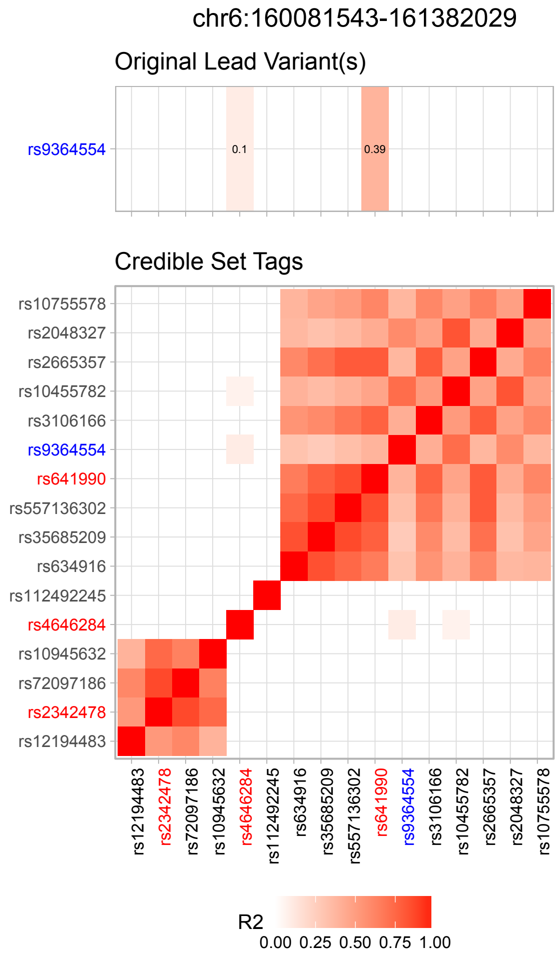

(h)

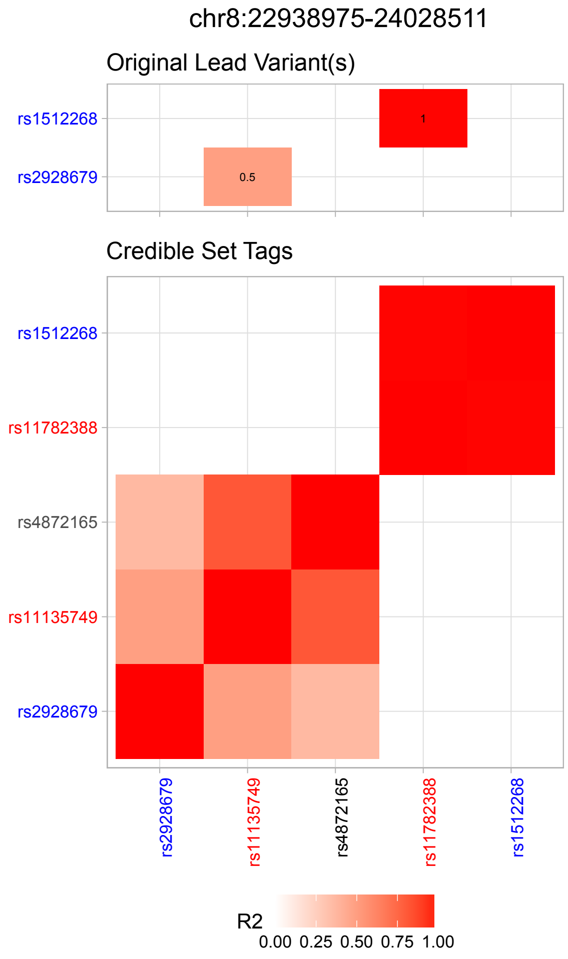

Supplementary Figure 6 (continued)

(i)

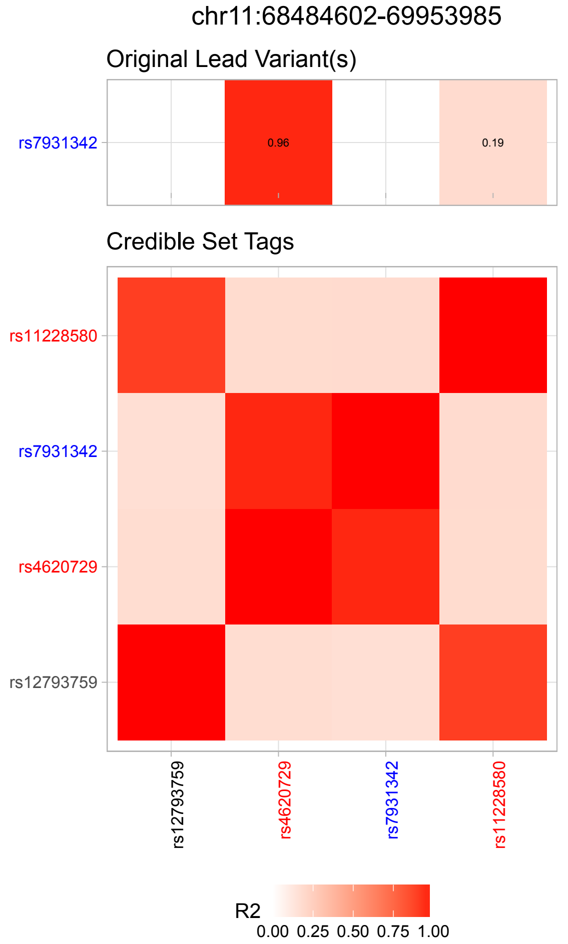

(j)

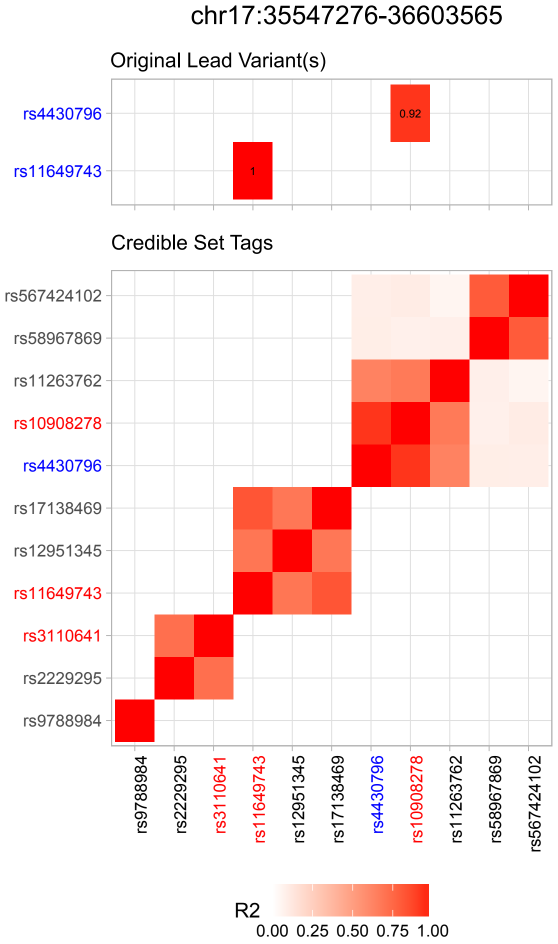

(k)

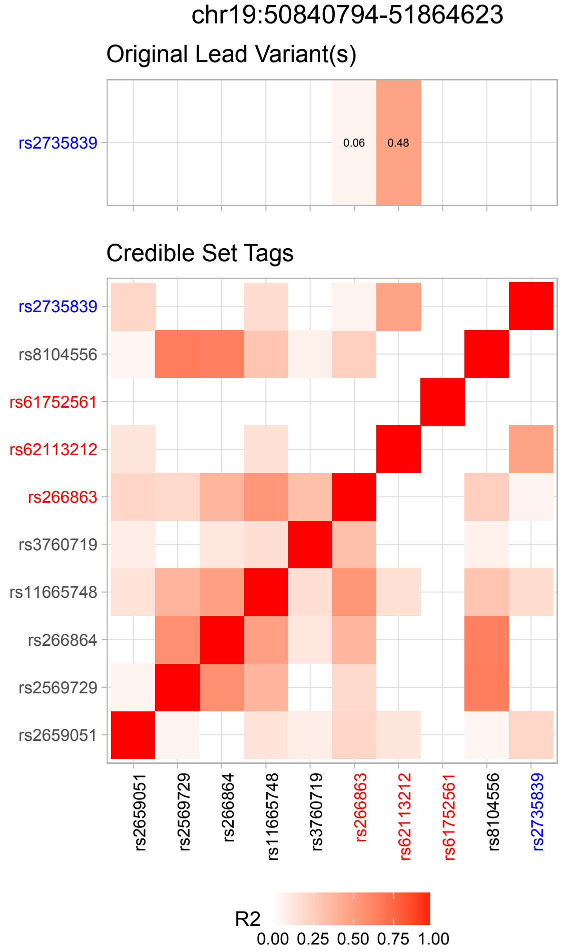

(l)

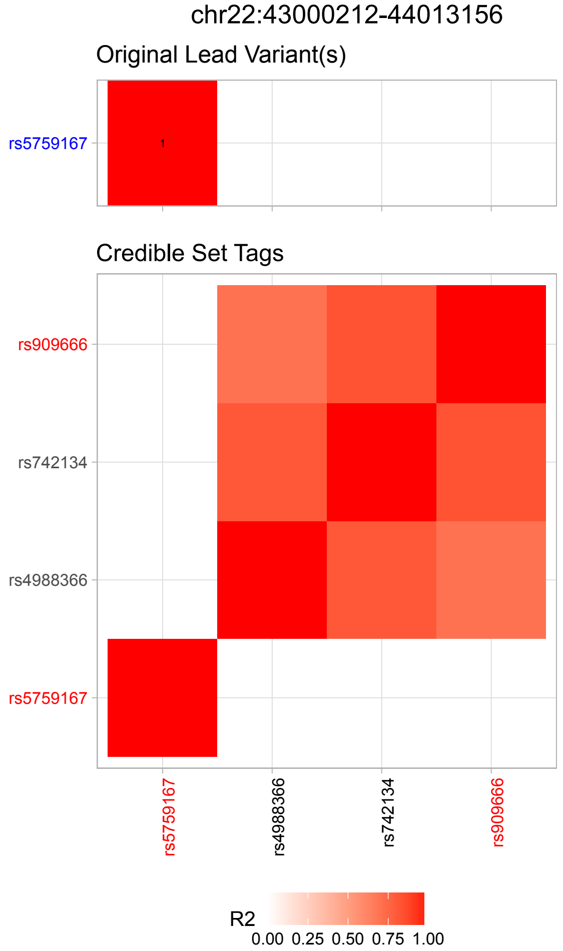

Supplementary Figure 6 – Heat-map plot depicting LD between the original index SNPs and tag variants included in the credible set for regions containing multiple independent signals. The 12 regions identified to contain multiple independent signals by JAM are depicted in separate panels **(a-l)**. Original index SNPs are denoted in blue and the combination of tags given the greatest posterior support by JAM, which were taken as representative variants for the individual independent signals during the FRR calculation, are shown in red. The Original Lead Variant(s) track shows LD between the original GWAS SNP(s) and FRR representative variants. The Credible Set Tags grid depicts LD between all tag variants selected in the credible set; the original index SNP is also included if it was not selected in the final credible set or was represented by another tag.

Supplementary Figure 7

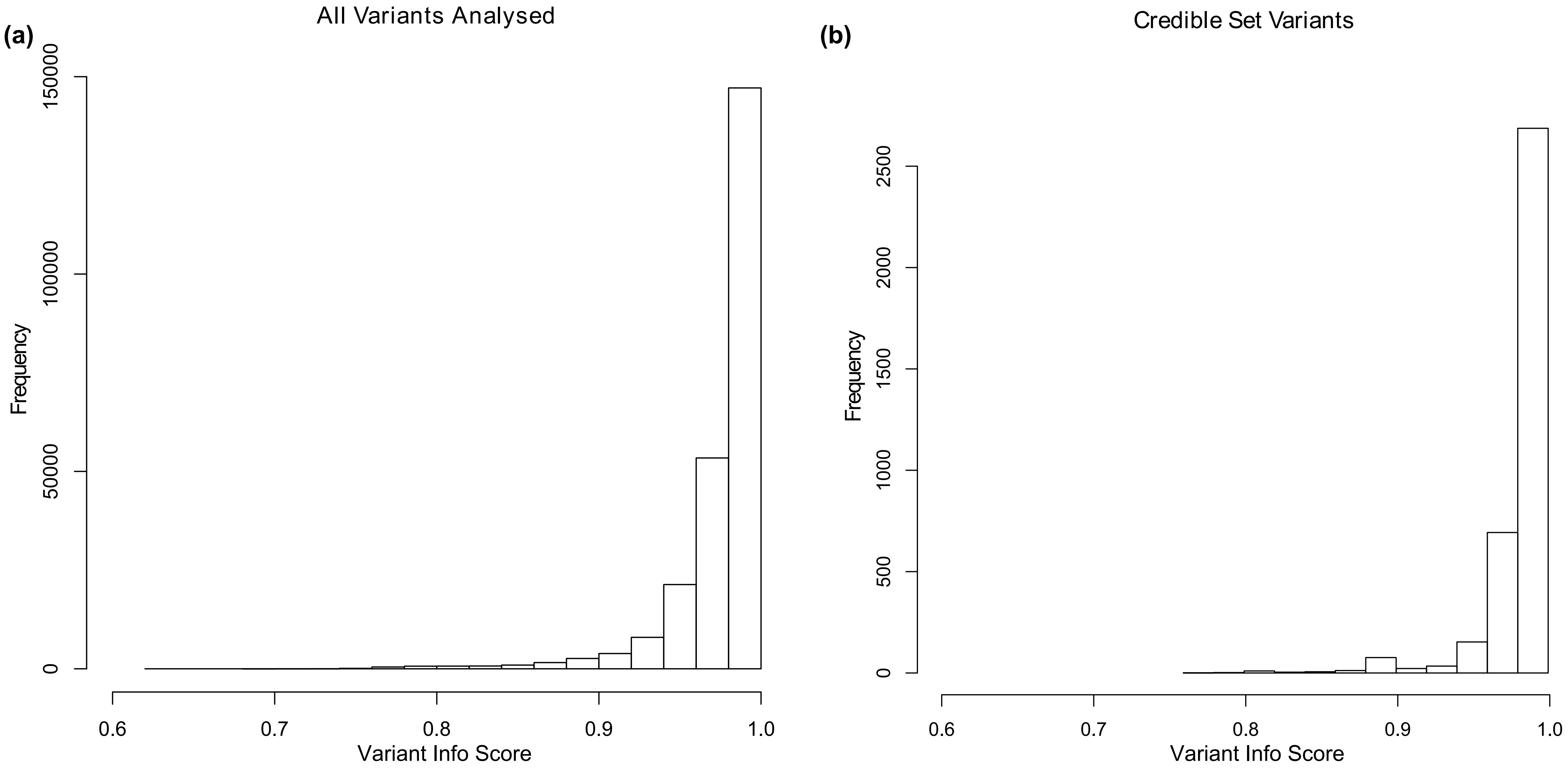

Supplementary Figure 7 – Histogram of variant Info scores in the imputed meta-analysis data after QC filtering. **(a)** All variants analysed during fine-mapping. **(b)** Variants within the 95% credible set selected by JAM.

Region Boundary	SNPs Mapped	Lowest *P* Value of any SNP in Region in EUR meta-analysis	Comments
chr1:10056097-11056097	rs636291 (*PEX14*)	3.88×10^{-7}	Previously reported for young onset PrCa only
chr9:123927373-125154402	rs1571801 (*DAB2IP*)	3.05×10^{-4}	Previously reported for aggressive PrCa only
chr14:60622526-61622526	rs7153648 (*SIX1*)	7.74×10^{-5}	Previously reported in a multi-ethnic meta-analysis only
chr16:71191329-72191329	rs12051443 (*PHLPP2*)	3.98×10^{-5}	Previously reported in a multi-ethnic meta-analysis only
chr19:54204670-55297848	rs103294 (*LILRA3*)	7.16×10^{-4}	Previously reported in a Chinese population only

Supplementary Table 1 – List of previously published PrCa GWAS associations that were not replicated in our European meta-analysis prior to fine-mapping. The previously reported association was considered replicated if any variant(s) within the assigned fine-mapping region boundary had a marginal *P*-value beyond the threshold specified for genome-wide significance of association with PrCa ($P<5\text{x}10^{-8}$).

	Beta-Binomial(1,P) Prior probability			
**Number of Variants in region (P)**	**No effect**	**≥1 effect**	**≥2 effects**	**≥3 effects**
5	0.5	0.5	0.22	0.08
10	0.5	0.5	0.24	0.11
100	0.5	0.5	0.25	0.12
1000	0.5	0.5	0.25	0.12

Supplementary Table 2 – Beta-binomial prior probabilities for different numbers of independent causal variants as a function of the number of variants in a region.

Supplementary Note 1 – Funding & Acknowledgements. Information and acknowledgements for consortia contributing to the meta-analysis and for individual study groups within the PRACTICAL Consortium.

GWAS Studies in the Meta-Analysis Dataset

The authors wish to pay tribute to Brian Henderson, who was a driving force behind the OncoArray project, for his vision and leadership, and sadly passed away before seeing its fruition.

<u>OncoArray</u>
Genotyping of the OncoArray was funded by the US National Institutes of Health (NIH) [U19 CA 148537 for ELucidating Loci Involved in Prostate cancer SuscEptibility (ELLIPSE) project and X01HG007492 to the Center for Inherited Disease Research (CIDR) under contract number HHSN268201200008I] and by Cancer Research UK grant A8197/A16565. Additional analytic support was provided by NIH NCI U01 CA188392 (PI: Schumacher).

This study would not have been possible without the contributions of the following: Coordination team, bioinformatician and genotyping centers

Genotyping at CCGE, Cambridge: Craig Luccarini, Caroline Baynes, Patricia Harrington and Don Conroy

<u>iCOGS</u>
Funding for the iCOGS infrastructure came from: the European Community's Seventh Framework Programme under grant agreement n° 223175 (HEALTH-F2-2009-223175) (COGS), Cancer Research UK (C1287/A10118, C1287/A 10710, C12292/A11174, C1281/A12014, C5047/A8384, C5047/A15007, C5047/A10692, C8197/A16565), the National Institutes of Health (CA128978) and Post-Cancer GWAS initiative (1U19 CA148537, 1U19 CA148065 and 1U19 CA148112 - the GAME-ON initiative), the Department of Defence (W81XWH-10-1-0341), the Canadian Institutes of Health Research (CIHR) for the CIHR Team in Familial Risks of Breast Cancer, Komen Foundation for the Cure, the Breast Cancer Research Foundation, and the Ovarian Cancer Research Fund.

This study would not have been possible without the contributions of the following: Per Hall (COGS); Douglas F. Easton, Paul Pharoah, Kyriaki Michailidou, Manjeet K. Bolla, Qin Wang (BCAC), Andrew Berchuck (OCAC), Rosalind A. Eeles, Douglas F. Easton, Ali Amin Al Olama, Zsofia Kote-Jarai, Sara Benlloch (PRACTICAL), Georgia Chenevix-Trench, Antonis Antoniou, Lesley McGuffog, Fergus Couch and Ken Offit (CIMBA), Joe Dennis, Alison M. Dunning, Andrew Lee, and Ed Dicks, Craig Luccarini and the staff of the Centre for Genetic Epidemiology Laboratory, Javier Benitez, Anna Gonzalez-Neira and the staff of the CNIO genotyping unit, Jacques Simard and Daniel C. Tessier, Francois Bacot, Daniel Vincent, Sylvie LaBoissière and Frederic Robidoux and the staff of the McGill University and Génome Québec Innovation Centre, Stig E. Bojesen, Sune F. Nielsen, Borge G. Nordestgaard, and the staff of the Copenhagen DNA laboratory, and Julie M. Cunningham, Sharon A. Windebank, Christopher A. Hilker, Jeffrey Meyer and the staff of Mayo Clinic Genotyping Core Facility

CRUK and PRACTICAL consortium
This work was supported by the Canadian Institutes of Health Research, European Commission's Seventh Framework Programme grant agreement n° 223175 (HEALTH-F2-2009-223175), Cancer Research UK Grants C5047/A7357, C1287/A10118, C1287/A16563, C5047/A3354, C5047/A10692, C16913/A6135, and The National Institute of Health (NIH) Cancer Post-Cancer GWAS initiative grant: No. 1 U19 CA 148537-01 (the GAME-ON initiative).

We would also like to thank the following for funding support: The Institute of Cancer Research and The Everyman Campaign, The Prostate Cancer Research Foundation, Prostate Research Campaign UK (now Prostate Action), The Orchid Cancer Appeal, The National Cancer Research Network UK, The National Cancer Research Institute (NCRI) UK. We are grateful for support of NIHR funding to the NIHR Biomedical Research Centre at The Institute of Cancer Research and The Royal Marsden NHS Foundation Trust.
The Prostate Cancer Program of Cancer Council Victoria also acknowledge grant support from The National Health and Medical Research Council, Australia (126402, 209057, 251533, , 396414, 450104, 504700, 504702, 504715, 623204, 940394, 614296,), VicHealth, Cancer Council Victoria, The Prostate Cancer Foundation of Australia, The Whitten Foundation, PricewaterhouseCoopers, and Tattersall's. EAO, DMK, and EMK acknowledge the Intramural Program of the National Human Genome Research Institute for their support.

BPC3
The BPC3 was supported by the U.S. National Institutes of Health, National Cancer Institute (cooperative agreements U01-CA98233 to D.J.H., U01-CA98710 to S.M.G., U01-CA98216 to E.R., and U01-CA98758 to B.E.H., and Intramural Research Program of NIH/National Cancer Institute, Division of Cancer Epidemiology and Genetics).

CAPS
CAPS GWAS study was supported by the Swedish Cancer Foundation (grant no 09-0677, 11-484, 12-823), the Cancer Risk Prediction Center (CRisP; www.crispcenter.org), a Linneus Centre (Contract ID 70867902) financed by the Swedish Research Council, Swedish Research Council (grant no K2010-70X-20430-04-3, 2014-2269)

PEGASUS
PEGASUS was supported by the Intramural Research Program, Division of Cancer Epidemiology and Genetics, National Cancer Institute, National Institutes of Health.

Additional Funding and Acknowledgments from Studies in the PRACTICAL Consortium

Information regarding the PRACTICAL consortium can be found at http://practical.icr.ac.uk

Aarhus

This study was supported by Innovation Fund Denmark, the Danish Cancer Society and The Velux Foundation (Veluxfonden).

The Danish Cancer Biobank (DCB) is acknowledged for biological material.

AHS

This work was supported by the Intramural Research Program of the NIH, National Cancer Institute, Division of Cancer Epidemiology and Genetics (Z01CP010119).

APCB

The Australian Prostate Cancer BioResource (APCB) was supported by The National Health and Medical Research Council, Enabling Grant [614296] and the Prostate Cancer Foundation of Australia.

The Australian Prostate Cancer BioResource (APCB) would like to acknowledge and sincerely thank the urologists, pathologists, coordinators, data managers, nurses and patient participants who have generously and altruistically supported the APCB.

W.D.T. was supported by The U.S. Department of Defense Prostate Cancer Research Program (Transformative Impact Award W81XWH-13-2-0093); The National Health and Medical Research Council (NHMRC) of Australia (1121057); the Prostate Cancer Foundation of Australia/Cancer Australia (1012337 and 1043482); Prostate Cancer Foundation of Australia/Movember Revolutionary Team Award; the Ray and Shirl Norman Cancer Research Trust.

ATBC

The ATBC Study is supported by the Intramural Research Program of the U.S. National Cancer Institute, National Institutes of Health, and by U.S. Public Health Service contract HHSN261201500005C from the National Cancer Institute, Department of Health and Human Services.

Canary PASS

PASS was supported by Canary Foundation and the National Cancer Institute's Early Detection Research Network) U01 CA086402)

CCI

This work was awarded by Prostate Cancer Canada and is proudly funded by the Movember Foundation - Grant # D2013-36.

The CCI group would like to thank David Murray, Razmik Mirzayans, and April Scott for their contribution to this work.

CHIPGECS

Orchid, Wuxi Second Hospital Research Funds, National Natural Science foundation of China for funding support to H Zhang (Grant No: 30671793 and 81072377), N Feng (Grant No: 81272831), SC Zhao (Grant No: 81072092 and 81328017)

This work was conducted on behalf of the CHIPGECS Consortia. We acknowledge the contribution of doctors, nurses and postgraduate research students at the CHIPGENCS sample collecting centers.

COH

SLN is partially supported by the Morris and Horowitz Families Endowed Professorship

COSM

The Swedish Research Council, the Swedish Cancer Foundation

CPCS1 & CPCS2

Department of Clinical Biochemistry, Herlev and Gentofte Hospital, Copenhagen University Hospital, Herlev Ringvej 75, DK-2730 Herlev, Denmark

We thank the participants and staff of the Copenhagen General Population Study for their important contributions.

CPS-II

The American Cancer Society funds the creation, maintenance, and updating of the Cancer Prevention Study II cohort.

We thank the CPS-II participants and Study Management Group for their invaluable contributions to this research. We would also like to acknowledge the contribution to this study from central cancer registries supported through the Centers for Disease Control and Prevention National Program of Cancer Registries, and cancer registries supported by the National Cancer Institute Surveillance Epidemiology and End Results program.

EPIC

The coordination of EPIC was financially supported by the European Commission (DG-SANCO) and the International Agency for Research on Cancer. The national cohorts (that recruited male participants) are supported by Danish Cancer Society (Denmark); German Cancer Aid, German Cancer Research Center (DKFZ), Federal Ministry of Education and Research (BMBF), Deutsche Krebshilfe, Deutsches Krebsforschungszentrum and Federal Ministry of Education and Research (Germany); the Hellenic Health Foundation (Greece); Associazione Italiana per la Ricerca sul Cancro-AIRC-Italy and National Research Council (Italy); Dutch Ministry of Public Health, Welfare and Sports (VWS), Netherlands Cancer Registry (NKR), LK Research Funds, Dutch Prevention Funds, Dutch ZON (Zorg Onderzoek Nederland), World Cancer Research Fund (WCRF), Statistics Netherlands (The Netherlands); Health Research Fund (FIS), PI13/00061 to Granada; , PI13/01162 to EPIC-Murcia), Regional Governments of Andalucía, Asturias, Basque Country, Murcia and Navarra, ISCIII RETIC (RD06/0020) (Spain); Swedish Cancer Society, Swedish Research Council and County Councils of Skåne and Västerbotten (Sweden); Cancer Research UK

(14136 to EPIC-Norfolk; C570/A16491 and C8221/A19170 to EPIC-Oxford), Medical Research Council
(1000143 to EPIC-Norfolk, MR/M012190/1 to EPIC-Oxford) (United Kingdom). For information on how to submit an application for gaining access to EPIC data and/or biospecimens, please follow the instructions at http://epic.iarc.fr/access/index.php

EPICAP

The EPICAP study was supported by grants from Ligue Nationale Contre le Cancer, Ligue départementale du Val de Marne; Fondation de France; Agence Nationale de sécurité sanitaire de l'alimentation, de l'environnement et du travail (ANSES)

The EPICAP study group would like to thank all urologists, Antoinette Anger and Hasina Randrianasolo (study monitors), Anne-Laure Astolfi, Coline Bernard, Oriane Noyer, Marie-Hélène De Campo, Sandrine Margaroline, Louise N'Diaye, Sabine Perrier-Bonnet (Clinical Research nurses).

ERSPC

This study was supported by the DutchCancerSociety(KWF94-869,98-1657,2002-277,2006-3518, 2010-4800); The Netherlands Organisation for HealthResearch and Development (ZonMW-002822820,22000106,50-50110-98-311, 62300035), The Dutch Cancer Research Foundation(SWOP), and an unconditional grant from Beckman-Coulter-HybritechInc.

ESTHER

The ESTHER study was supported by a grant from the Baden Württemberg Ministry of Science, Research and Arts.

The ESTHER group would like to thank Hartwig Ziegler, Sonja Wolf, Volker Hermann, Heiko Müller, Karina Dieffenbach, Katja Butterbach for valuable contributions to the study.

FHCRC

The FHCRC studies were supported by grants R01-CA056678, R01-CA082664, and R01-CA092579 from the US National Cancer Institute, National Institutes of Health, with additional support from the Fred Hutchinson Cancer Research Center.

We thank all the men who participated in these studies.

Gene-PARE

The Gene-PARE study was supported by grants 1R01CA134444 from the U.S. National Institutes of Health , PC074201 and W81XWH-15-1-0680 from the Prostate Cancer Research Program of the Department of Defense and RSGT-05-200-01-CCE from the American Cancer Society. S.L.K. is supported by 1K07CA187546 from the U.S. National Cancer Institute.

HPFS

The Health Professionals Follow-up Study was supported by grants UM1CA167552, CA133891, CA141298, and P01CA055075.

We are grateful to the participants and staff of the Physicians' Health Study and Health Professionals Follow-Up Study for their valuable contributions, as well as the following state

cancer registries for their help: AL, AZ, AR, CA, CO, CT, DE, FL, GA, ID, IL, IN, IA, KY, LA, ME, MD, MA, MI, NE, NH, NJ, NY, NC, ND, OH, OK, OR, PA, RI, SC, TN, TX, VA, WA, WY.

IMPACT
The IMPACT study was funded by The Ronald and Rita McAulay Foundation, CR-UK Project grant (C5047/A1232), Cancer Australia, AICR Netherlands A10-0227, Cancer Australia and Cancer Council Tasmania, NIHR, EU Framework 6, Cancer Councils of Victorial and South Australia, Philanthropic donation to Northshore University Health System. We acknowledge support from the National Institute for Health Research (NIHR) to the Biomedical Research Centre at The Institute of Cancer Research and Royal Marsden Foundation NHS Trust.

We acknowledge the IMPACT study steering committee, collaborating centres and participants.

IPO-Porto
The IPO-Porto study was funded by Fundação para a Ciência e a Tecnologia (FCT; UID/DTP/00776/2013 and PTDC/DTP-PIC/1308/2014) and by IPO-Porto Research Center (CI-IPOP-16-2012 and CI-IPOP-24-2015). MC and MPS are research fellows from Liga Portuguesa Contra o Cancro, Núcleo Regional do Norte. SM is a research fellow from FCT (SFRH/BD/71397/2010).

We would like to express our gratitude to all patients and families who have participated in this study.

KULEUVEN
F.C. and S.J. are holders of grants from FWO Vlaanderen (G.0684.12N and G.0830.13N), the Belgian federal government (National Cancer Plan KPC_29_023), and a Concerted Research Action of the KU Leuven (GOA/15/017). TVDB is holder of a doctoral fellowship of the FWO.

Malaysia
The study was funded by the University Malaya High Impact Research Grant (HIR/MOHE/MED/35 to A.R.).

We thank all associates in the Urology Unit, University of Malaya, Cancer Research Malaysia (CRM) and the Malaysian Men's Health Initiative (MMHI).

MAYO
The Mayo group was supported by the US National Cancer Institute (R01CA72818)

MCCS
The Melbourne Collaborative Cohort Study (MCCS) cohort recruitment was funded by VicHealth and Cancer Council Victoria. The MCCS was further supported by Australian National Health and Medical Research Council grants 209057 and 396414 and by infrastructure provided by Cancer Council Victoria. Cases and their vital status were ascertained through the Victorian Cancer Registry and the Australian Institute of Health and Welfare, including the National Death Index and the Australian Cancer Database.

MCC-Spain
The study was partially funded by the "Accion Transversal del Cancer", approved on the Spanish Ministry Council on the 11th October 2007, by the Instituto de Salud Carlos III-FEDER (PI08/1770, PI09/00773-Cantabria, PI11/01889-FEDER, PI12/00265, PI12/01270, PI12/00715, PI15/00069), by the Fundación Marqués de Valdecilla (API 10/09), by the Spanish Association Against Cancer (AECC) Scientific Foundation and by the Catalan Government DURSI grant 2009SGR1489. Samples: Biological samples were stored at the Parc de Salut MAR Biobank (MARBiobanc; Barcelona) which is supported by Instituto de Salud Carlos III FEDER (RD09/0076/00036). Also sample collection was supported by the Xarxa de Bancs de Tumors de Catalunya sponsored by Pla Director d'Oncologia de Catalunya (XBTC). ISGlobal is a member of the CERCA Programme, Generalitat de Catalunya.

We acknowledge the contribution from Esther Gracia-Lavedan in preparing the data. We thank all the subjects who participated in the study and all MCC-Spain collaborators.

MEC
The MEC was supported by NIH grants CA63464, CA54281, CA098758, and CA164973.

MOFFITT
The Moffitt group was supported by the US National Cancer Institute (R01CA128813, PI: J.Y. Park).

Oslo
CONOR was supported by grants from the Nordic Cancer Union, the Swedish Cancer Society (2012/823) and the Swedish Research Council (2014/2269).

The authors wish to acknowledge the services of CONOR, the contributing research centres delivering data to CONOR, and all the study participants.

PCMUS
The PCMUS study was supported by the Bulgarian National Science Fund, Ministry of Education and Science (contract DOO-119/2009; DUNK01/2-2009; DFNI-B01/28/2012) with additional support from the Science Fund of Medical University - Sofia (contract 51/2009; 8I/2009; 28/2010;).

PHS
The Physicians' Health Study was supported by grants CA34944, CA40360, CA097193, HL26490 and HL34595.

We are grateful to the participants and staff of the Physicians' Health Study and Health Professionals Follow-Up Study for their valuable contributions, as well as the following state cancer registries for their help: AL, AZ, AR, CA, CO, CT, DE, FL, GA, ID, IL, IN, IA, KY, LA, ME, MD, MA, MI, NE, NH, NJ, NY, NC, ND, OH, OK, OR, PA, RI, SC, TN, TX, VA, WA, WY.

PLCO
This PLCO study was supported by the Intramural Research Program of the Division of Cancer Epidemiology and Genetics, National Cancer Institute, NIH

The authors thank Drs. Christine Berg and Philip Prorok, Division of Cancer Prevention at the National Cancer Institute, the screening center investigators and staff of the PLCO Cancer Screening Trial for their contributions to the PLCO Cancer Screening Trial. We thank Mr. Thomas Riley, Mr. Craig Williams, Mr. Matthew Moore, and Ms. Shannon Merkle at Information Management Services, Inc., for their management of the data and Ms. Barbara O'Brien and staff at Westat, Inc. for their contributions to the PLCO Cancer Screening Trial. We also thank the PLCO study participants for their contributions to making this study possible.

PRAGGA

PRAGGA was supported by Programa Grupos Emergentes, Cancer Genetics Unit, CHUVI Vigo Hospital, Instituto de Salud Carlos III, Spain.

PRAGGA wishes to thank Victor Muñoz Garzón, Manuel Enguix Castelo, Sara Miranda Ponte, Carmen M Redondo, Manuel Calaza, Francisco Gude Sampedro, Joaquín González-Carreró and the staff of the Department of Pathology and Biobank of University Hospital Complex of Vigo, Instituto de Investigación Sanitaria Galicia Sur (IISGS), SERGAS, Vigo, Spain; Máximo Fraga, José Antúnez and the Biobank of University Hospital Complex of Santiago, Santiago de Compostela, Spain; and Maria Torres, Angel Carracedo and the Galician Foundation of Genomic Medicine.

PROCAP

PROCAP was supported by the Swedish Cancer Foundation (08-708, 09-0677).

We thank and acknowledge all of the participants in the PROCAP study. We thank Carin Cavalli-Björkman and Ami Rönnberg Karlsson for their dedicated work in the collection of data. Michael Broms is acknowledged for his skilful work with the databases. KI Biobank is acknowledged for handling the samples and for DNA extraction. We acknowledge The NPCR steering group: Pär Stattin (chair), Anders Widmark, Stefan Karlsson, Magnus Törnblom, Jan Adolfsson, Anna Bill-Axelson, Ove Andrén, David Robinson, Bill Pettersson, Jonas Hugosson, Jan-Erik Damber, Ola Bratt, Göran Ahlgren, Lars Egevad, and Roy Ehrnström.

PROFILE

We would like to acknowledge the support of the Ronald and Rita McAulay Foundation and Cancer Research UK. We also acknowledge support from the National Institute for Health Research (NIHR) to the Biomedical Research Centre at The Institute of Cancer Research and Royal Marsden Foundation NHS Trust

We acknowledge the Profile study steering committee and participants.

PROGReSS

The PROGReSS study is funded by grants from the Spanish Ministry of Health (INT15/00070; INT16/00154; FIS PI10/00164, FIS PI13/02030; FIS PI16/00046); the Spanish Ministry of Economy and Competitiveness (PTA2014-10228-I) and Fondo Europeo de Desarrollo Regional (FEDER 2007-2013). LF was funded by European Union Marie Sklodowska-Curie Individual Fellowship (MSCA-IF-2014-EF-656144)

ProMPT & ProtecT

ProtecT would like to acknowledge the support of The University of Cambridge, Cancer Research UK. Cancer Research UK grants [C8197/A10123] and [C8197/A10865] supported the genotyping team. We would also like to acknowledge the support of the National Institute for Health

Research which funds the Cambridge Bio-medical Research Centre, Cambridge, UK. We would also like to acknowledge the support of the National Cancer Research Prostate Cancer: Mechanisms of Progression and Treatment (PROMPT) collaborative (grant code G0500966/75466) which has funded tissue and urine collections in Cambridge. We are grateful to staff at the Welcome Trust Clinical Research Facility, Addenbrooke's Clinical Research Centre, Cambridge, UK for their help in conducting the ProtecT study. We also acknowledge the support of the NIHR Cambridge Biomedical Research Centre, the DOH HTA (ProtecT grant) and the NCRI / MRC (ProMPT grant) for help with the bio-repository. The UK Department of Health funded the ProtecT study through the NIHR Health Technology Assessment Programme (projects 96/20/06, 96/20/99). The ProtecT trial and its linked ProMPT and CAP (Comparison Arm for ProtecT) studies are supported by Department of Health, England; Cancer Research UK grant number C522/A8649, Medical Research Council of England grant number G0500966, ID 75466 and The NCRI, UK. The epidemiological data for ProtecT were generated though funding from the Southwest National Health Service Research and Development. DNA extraction in ProtecT was supported by USA Dept of Defense award W81XWH-04-1-0280, Yorkshire Cancer Research and Cancer Research UK. The authors would like to acknowledge the contribution of all members of the ProtecT study research group. Richard Martin was supported by a Cancer Research UK Programme Grant (C18281/A19169) and the National Institute for Health Research Bristol Nutrition Biomedical Research Centre based at University Hospitals Bristol NHS Foundation Trust and the University of Bristol. The views and opinions expressed therein are those of the authors and do not necessarily reflect those of the Department of Health of England. The bio-repository from ProtecT is supported by the NCRI (ProMPT) Prostate Cancer Collaborative and the Cambridge BMRC grant from NIHR. We thank the National Institute for Health Research, Hutchison Whampoa Limited, the Human Research Tissue Bank (Addenbrooke's Hospital), and Cancer Research UK. The authors would like to thank those men with prostate cancer and the subjects who have donated their time and their samples to the Cambridge Biorepository, which were used in this research. We also would like to acknowledge to support of the research staff in S4 who so carefully curated the samples and the follow-up data (Jo Burge, Marie Corcoran, Anne George, and Sara Stearn).

QLD
The QLD research is supported by The National Health and Medical Research Council (NHMRC) Australia Project Grants [390130, 1009458] and NHMRC Career Development Fellowship and Cancer Australia PdCCRS funding to J Batra.

The QLD team would like to acknowledge and sincerely thank the urologists, pathologists, data managers and patient participants who have generously and altruistically supported the QLD cohort.

RAPPER
RAPPER is funded by Cancer Research UK [C1094/A11728; C1094/A18504] and Experimental Cancer Medicine Centre funding [C1467/A7286]

The RAPPER group thank Rebecca Elliott for project management.

SEARCH
SEARCH is funded by a programme grant from Cancer Research UK [C490/A10124] and supported by the UK National Institute for Health Research Biomedical Research Centre at the

University of Cambridge. The University of Cambridge has received salary support in respect of PP from the NHS in the East of England through the Clinical Academic Reserve.

SFPCS

SFPCS was funded by the California Cancer Research Fund grant 99-00527V-10182.

SNP_Prostate_Ghent

The study was supported by the National Cancer Plan, financed by the Federal Office of Health and Social Affairs, Belgium.

SPAG

Wessex Medical Research

Hope for Guernsey, MUG, HSSD, MSG, Roger Allsopp

STHM2

STHM2 was supported by grants from The Strategic Research Programme on Cancer (StratCan), Karolinska Institutet; the Linné Centre for Breast and Prostate Cancer (CRISP, number 70867901), Karolinska Institutet; The Swedish Research Council (number K2010-70X-20430-04-3) and The Swedish Cancer Society (numbers 11-0287 and 11-0624); Stiftelsen Johanna Hagstrand och Sigfrid Linnérs minne; Swedish Council for Working Life and Social Research (FAS), number 2012-0073.

The authors acknowledge the Karolinska University Laboratory, Aleris Medilab, Unilabs and the Regional Prostate Cancer Registry for performing analyses and help to retrieve data. Carin Cavalli–Björkman and Britt-Marie Hune for their enthusiastic work as research nurses. Astrid Björklund for skilful data management. We wish to thank the BBMRI.se biobank facility at Karolinska Institutet for biobank services.

SWOG-PCPT

PCPT is funded by Public Health Service grants U10CA37429 and 5UM1CA182883 from the National Cancer Institute.

The authors thank the site investigators and staff and, most importantly, the participants from PCPT who donated their time to this trial.

SWOG-SELECT

SELECT is funded by Public Health Service grants U10CA37429 and 5UM1CA182883 from the National Cancer Institute.

The authors thank the site investigators and staff and, most importantly, the participants from SELECT who donated their time to this trial.

TAMPERE

The Tampere (Finland) study was supported by the Academy of Finland (251074), The Finnish Cancer Organisations, Sigrid Juselius Foundation, and the Competitive Research Funding of the Tampere University Hospital (X51003). The PSA screening samples were collected by the Finnish part of ERSPC (European Study of Screening for Prostate Cancer).

TAMPERE would like to thank Riina Liikanen, Liisa Maeaettaenen and Kirsi Talala for their work on samples and databases.

Toronto

Prostate Cancer Canada Movember Discovery Grant (D2013-17) to RJH; Canadian Cancer Society Research Institute Career Development Award in Cancer Prevention (2013-702108) to RJH

UKGPCS

UKGPCS would also like to thank the following for funding support: The Institute of Cancer Research and The Everyman Campaign, The Prostate Cancer Research Foundation, Prostate Research Campaign UK (now Prostate Action), The Orchid Cancer Appeal, The National Cancer Research Network UK, The National Cancer Research Institute (NCRI) UK. We are grateful for support of NIHR funding to the NIHR Biomedical Research Centre at The Institute of Cancer Research and The Royal Marsden NHS Foundation Trust. UKGPCS should also like to acknowledge the NCRN nurses, data managers and Consultants for their work in the UKGPCS study.

UKGPCS would like to thank all urologists and other persons involved in the planning, coordination, and data collection of the study.

ULM

The Ulm group received funds from the German Cancer Aid (Deutsche Krebshilfe).

UTAH

The Keith and Susan Warshaw Fund, C. S. Watkins Urologic Cancer Fund and The Tennity Family Fund supported the Utah study. The project was supported by Award Number P30CA042014 from the National Cancer Institute

WUGS/WUPCS

WUGS would like to thank the following for funding support: The Anthony DeNovi Fund, the Donald C. McGraw Foundation, and the St. Louis Men's Group Against Cancer.
