# Supplementary material for: Fine-mapping of prostate cancer susceptibility loci in a large meta-analysis identifies candidate causal variants
Source: Nat Commun. 2018 Jun 11;9:2256. doi: 10.1038/s41467-018-04109-8 (PMC5995836; doi:10.1038/s41467-018-04109-8)
Supplement: Supplementary file 3 — Description of Additional Supplementary Files [file 41467_2018_4109_MOESM3_ESM.pdf]

Description of Additional Supplementary Files

Supplementary Data 1 – (a) Full list of variants within the 95% credible set selected by JAM. Statistical results after fine-mapping and from the original meta-analysis are provided, alongside detailed variant annotations. **(b)** List of all variants that had a marginal *P*-value beyond the threshold specified for genome-wide significance of association with PrCa ($P<5x10^{-8}$) in the 5 regions for which JAM was unable to resolve candidate variants, alongside detailed variant annotations.

Supplementary Data 2 – Enrichment of annotations among tags within the 95% credible set selected by JAM across all 75 regions included in this analysis. All tests for enrichment were performed upon the set of priority pruner tags, with binary annotations for all respective proxy variants inherited by their tag. **(a)** Joint modelling of all selected annotations using conditional quantile regression. **(b)** Fisher's exact test conducted for each annotation separately.

Supplementary Data 3 – Summary of regions in which variants selected in the 95% credible set selected by JAM, or variants that had a marginal *P*-value beyond the threshold specified for genome-wide significance of association with PrCa ($P<5x10^{-8}$) in the 5 regions in which JAM did not resolve candidate variants, were also identified as eQTLs in TCGA prostate adenocarcinoma data. The genes for which expression is altered are listed, alongside the numbers of variants in the JAM credible set and those for which a significantly associated, co-localised eQTL were detected.

Supplementary Data 4 – List of representative lead variants and their effect estimates used to calculate the proportion of familial relative risk (FRR) of PrCa explained. 'Onco Results (84 SNPs)' refer to the 84 EUR replicated original PrCa index SNPs used to calculate the FRR explained by these loci before fine-mapping. 'JAM Results (84 SNPs)' relates to the restricted subset of 84 'best' lead variants identified after fine-mapping and was used to estimate the change in proportion of FRR explained through replacement of index SNPs alone. 'JAM Results (99 SNPs)' relates to the full set of lead variants representing all 99 signals identified through fine-mapping and was used to estimate the total change in proportion of FRR explained after fine-mapping of these GWAS loci.

Supplementary Data 5 – Comparison between the results of our fine-mapping analysis and a previous study using data from the iCOGS project. Only the 48 regions that replicated with any variant at genome-wide significance in both studies are shown. Comparisons are provided for the number of signals and size of the credible set of candidate variants reported in each analysis.
